# The Impact of the Geologic History and Paleoclimate on the Diversification of East African Cichlids

**DOI:** 10.1155/2012/574851

**Published:** 2012-07-19

**Authors:** Patrick D. Danley, Martin Husemann, Baoqing Ding, Lyndsay M. DiPietro, Emily J. Beverly, Daniel J. Peppe

**Affiliations:** ^1^Department of Biology, Baylor University, One Bear Place no. 97388, Waco, TX 76798, USA; ^2^Department of Geology, Baylor University, One Bear Place no. 97388, Waco, TX 76798, USA

## Abstract

The cichlid fishes of the East African Great Lakes are the largest extant vertebrate radiation identified to date. These lakes and their surrounding waters support over 2,000 species of cichlid fish, many of which are descended from a single common ancestor within the past 10 Ma. The extraordinary East African cichlid diversity is intricately linked to the highly variable geologic and paleoclimatic history of this region. Greater than 10 Ma, the western arm of the East African rift system began to separate, thereby creating a series of rift basins that would come to contain several water bodies, including the extremely deep Lakes Tanganyika and Malawi. Uplifting associated with this rifting backponded many rivers and created the extremely large, but shallow Lake Victoria. Since their creation, the size, shape, and existence of these lakes have changed dramatically which has, in turn, significantly influenced the evolutionary history of the lakes' cichlids. This paper reviews the geologic history and paleoclimate of the East African Great Lakes and the impact of these forces on the region's endemic cichlid flocks.

## 1. Introduction

East Africa had a highly dynamic geological and ecological history. Over the past 35 million years (Ma), tectonic plates have shifted, rifts in the landscape have opened, rivers have reversed course, and lakes have formed and desiccated. It is within this environment that the world's largest extant vertebrate radiation has originated. Centered within the East African Great Lakes, over 2,000 species of cichlid fish have diversified to fill nearly every niche available to a freshwater fish. All of these fish are endemic to East Africa, many are single lake endemics, and several are microendemics found only at isolated areas within a given lake. Here, we examine the geologic and climatic history of East Africa and discuss how these forces have influenced this spectacular vertebrate radiation.

### 1.1. Geologic Setting and East African Climate

**Figure 1:**
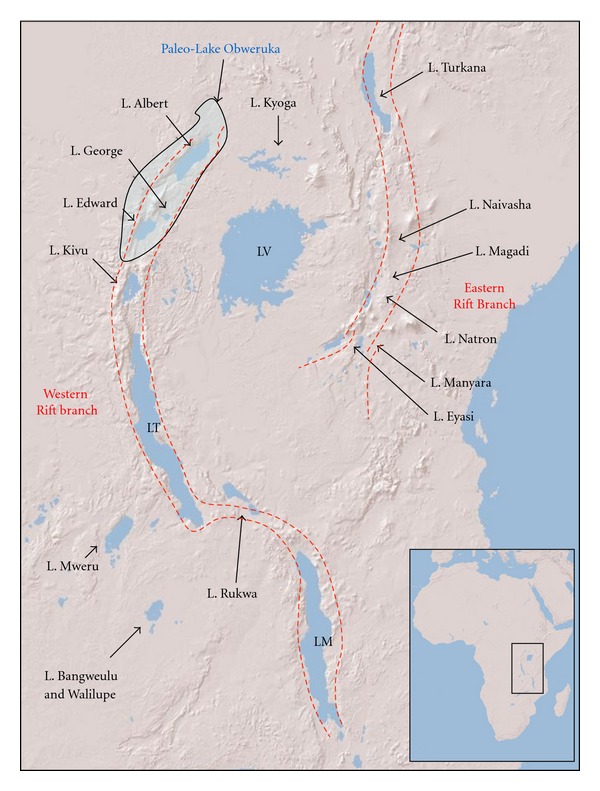
Geographic position of the study region and location of the East African rift. The position of paleo-Lake Obweruka is displayed in light blue [1]. The approximate locations of the two main branches of the East African rift system are displayed in red-dashed line (LV: Lake Victoria, LT: Lake Tanganyika, and LM: Lake Malawi).

The East Africa rift system (EARS) is the roughly north-south alignment of rift basins in East Africa (Figure 1) that defines the boundary between the Somalian and African plates [2, 3]. The EARS is divided into two structural branches that are also oriented roughly north-south (Figure 1). Rifting in the eastern branch began ~30–35 Ma in the Afar and Ethiopian Plateau and propagated north-south until it impinged on the strong Precambrian Tanzanian cratonic block, which is in the center of the East Africa Plateau [4]. The extensional stress associated with the rifting or with widespread plume-related uplift was then transported westward across the craton to weaker mobile crust on the craton's western edge creating the western branch of the rift [4, 5]. The timing of the initiation of the western branch of the EARS is uncertain and has been suggested to have begun as early as ~25 Ma to as recently as ~12–10 Ma [4, 5]. After its onset, rifting then continued to propagate in the western branch of the EARS forming the rift basins that encompass Lakes Tanganyika and Malawi [2–7]. Extension and uplift associated with rifting created a reversal in rivers flowing westward across the East African Plateau and caused backponding into a topographic low in between the two branches of the rift, forming Lake Victoria [6–12].

**Figure 2:**
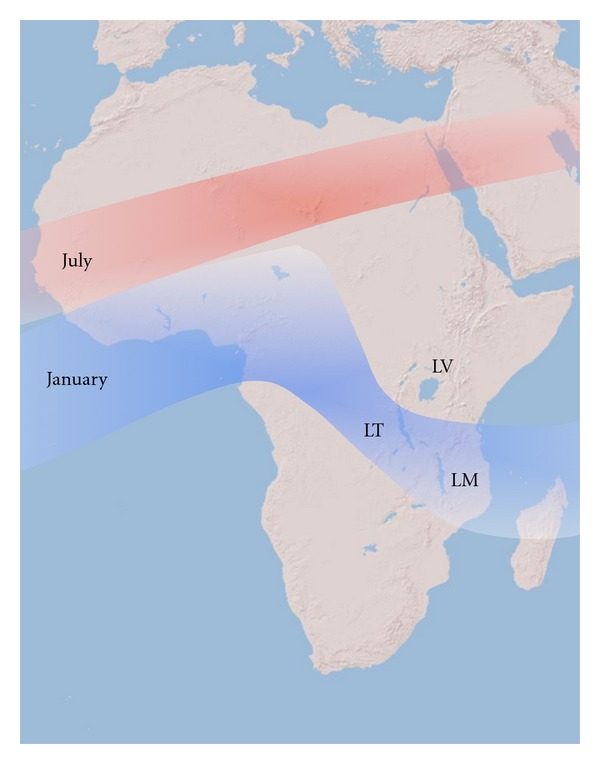
Seasonal position of the intertropical convergence zone (ITCZ). LV: Lake Victoria, LM: Lake Malawi, and LT: Lake Tanganyika.

The climate of the African Great Lakes (Lakes Tanganyika, Malawi, and Victoria) is driven primarily by annual changes in precipitation associated with the migration of the intertropical convergence zone (ITCZ) (Figure 2). The ITCZ is the zone of maximum insolation received by the Earth's surface and seasonally migrates between Tropics of Cancer and Capricorn in June and December, respectively. The warm air in the region of maximum heating rises, drawing the cooler trade winds equatorward, where they converge, effectively increasing convection and rainfall at the location of the ITCZ. The movement of the ITCZ across the African continent results in a wet-dry monsoonal climate for the African Great Lakes. The southernmost extent of the ITCZ is south of Lake Tanganyika; thus, it crosses the lake twice between September and May as it migrates southward to the Tropic of Capricorn and then back northward towards the Tropic of Cancer. As a result, the lake experiences a long wet season during which there is a lull in precipitation in January and February [31] when the core of the ITCZ is south of Lake Tanganyika and a pronounced, shorter dry season. The ITCZ crosses Lake Malawi once a year producing pronounced wet and dry seasons. The ITCZ crosses Lake Victoria twice due to the lake's position on the equator, resulting in two wet and two dry seasons. In each of the African Great Lakes, a significant portion of water loss is a result of evaporation; thus, the lakes are very sensitive to changes in precipitation. Variations in the position and intensity of the ITCZ can affect the duration of the wet seasons in each lake, causing aridity and significant changes in lake level [14, 15, 32].

### 1.2. East African Paleoclimate (10 Ma–Present)

The East African climate has been and continues to be dynamic [33]. Late Miocene (~8–10 Ma) climate in East Africa was humid and supported a variety of savanna and forest habitats, including rain forests [34]. Following this humid period, from ~7–5 Ma, the ice volume of the Antarctic ice sheet expanded and global temperatures fell [35–38]. This time period is also associated with aridification across East Africa [39, 40], as well as the uplift of the Himalayas and the resulting intensification of the Indian Monsoon [41], which may also have contributed to increased aridity. The early Pliocene (5–3 Ma) is characterized by warmer and wetter conditions globally and across Africa [42–46]. During this global warm, wet period, East Africa was also very humid [44], perhaps driving the expansion of Lake Tanganyika during the middle Pliocene [47]. Significant Northern Hemisphere Glaciation began and intensified between 3.2 and 2.6 Ma [48, 49] and beginning at ~2.0 Ma, Southern Hemisphere Glaciation expanded [50]. The interval beginning at ~2.8 Ma represents the onset of the glacial-interglacial cycles that characterizes the Pleistocene [51–53].

**Figure 3:**
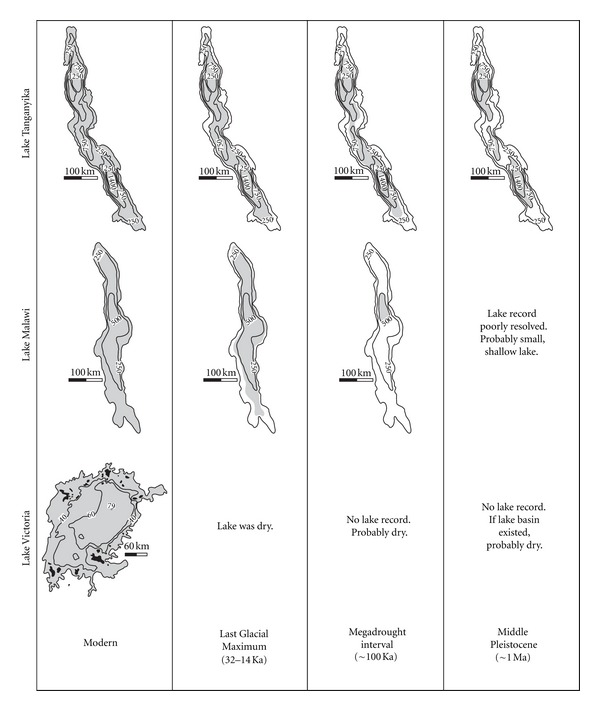
Bathymetric maps of Lakes Tanganyika, Malawi, and Victoria for modern, Last Glacial Maximum (15–32 ka), megadrought period (~100 ka), and the middle Pleistocene (~1 Ma). Reconstruction for ~1 Ma is based on data from Lake Tanganyika's subbasin [13], which has been extrapolated to the rest of the lake. Thus, this reconstruction is speculative and must be verified by additional data from the central and southern subbasins of Lake Tanganyika. Shaded areas show the maximum extent of lake during each interval. Lake Victoria's islands are shown in black. Lake levels based on [10, 13–22]. Bathymetric maps based on data from the World Lake Database (http://wldb.ilec.or.jp/).

Simultaneous with this onset of bipolar glaciation and glacial-interglacial cyclity is a cyclic trend in aridity across Africa [53, 61–64]. In particular, the climate in East Africa during the last 500 thousand years (ka) has been extremely variable transitioning between wet and dry intervals that have caused significant fluctuations in the lake levels of the African Great Lakes (Figure 3) [10, 13–18, 37, 65–71]. Of particular importance for cichlid populations is an interval from 135 to 70 ka when there were at least two intervals of extreme aridity, called megadroughts, during which lake levels in Lakes Malawi and Tanganyika probably fell dramatically, and Lake Victoria was likely completely desiccated (Figure 3) [15, 17–19]. Following this megadrought interval, climate variability decreased considerably [19]. During the Last Glacial Maximum (LGM), ~20–15 ka, Africa again experienced an increase in aridity, which caused the complete desiccation of Lake Victoria, a significant drop in lake level of Lake Tanganyika (~250–300 m), and only a relatively minor drop in the lake levels of Lake Malawi [9, 12, 15, 17, 19, 20, 31, 64, 65, 70, 71]. The Holocene represents an interval of a moderately fluctuating climate during which there have been modest fluctuations in the lake levels of the African Great Lakes [21, 47, 72].

### 1.3. East African Cichlids

**Figure 4:**
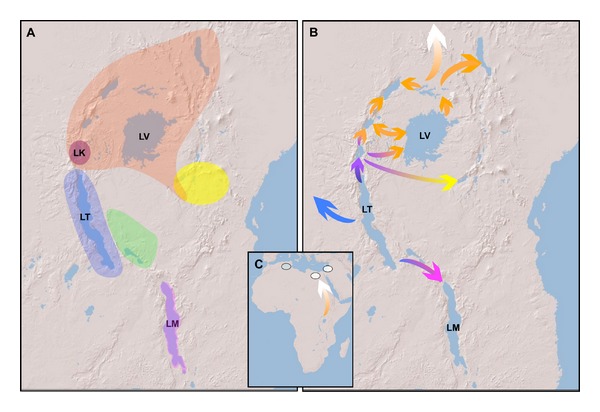
(A) The distribution of phylogenetic lineages. Colors indicate the distribution of genetic lineages: the distribution of the Lake Malawi lineage is displayed in purple, the Lake Tanganyika lineage is shaded in dark blue, the Malagarasi and Rukwa lineage are shown in green, the Lake Kivu lineage is colored purple, light red shading indicates the distribution of the Lake Victoria Superflock (LVSF), and the distribution of the South Kenyan—North Tanzanian lineage is displayed in yellow. (B) The possible colonization scenario for East African cichlids; the color of the arrows coincides with the colors of the lineages illustrated in part (A). (C) The distribution and possible colonization pathway for the North African and Israeli outposts of the LVSF. Phylogenetic data and colonization pathways are based on data from [23–30] and modified from a Figure  4(a) of [23].

Few taxa have been as influenced by the environmental and geological history of this region than fishes in the family Cichlidae. Cichlids are believed to have originated 121–165 Ma [73] within the Gondwanal supercontinent. Their Gondwanan origin is reflected in the current distribution of cichlids [74]: cichlids can be found throughout Africa, the Neotropics, and Madagascar with several additional species occurring in the Middle East, India, and Sri Lanka. With an estimated 3,000 species, cichlids are the most species-rich teleost family, and the focus of this extraordinary species diversity is the East African Great Lakes (Table 1, Figure 4).

An estimated 2,000 species of cichlids occur in Lakes Victoria, Tanganyika, and Malawi, the majority of which are believed to have diverged within the past 10 Ma [75]. Many of these species are narrow endemics that are not found outside the lake (or a location within the lake) in which they exist. This extraordinarily rapid, recent, and extensive species radiation has been shaped by the environmental and geological features that have affected the age, depth, and patterns of connectivity of the waters in which the cichlids diversified.

The aim of this paper is to synthesize the current understanding of the relationships between paleoclimate, geology, and the diversification of the East African cichlid species flock. Below, we explore these relationships in each of the lakes. In doing so, we hope to summarize the evolutionary diversification of East African cichlids within the context of the environmental and geological factors that have shaped their divergence.

## 2. Lake Tanganyika

### 2.1. Paleoclimate and Geologic History of Lake Tanganyika

Lake Tanganyika sits within the annual migration path of the ITCZ [76] and thus experiences both a rainy season (September–May) and a dry season (June–August), as well as changing wind direction and strength throughout the year [77]. Mean annual precipitation (MAP) in the region is ~1200 mm/yr [78] across most of the lake except on the eastern margins near the Mahale Mountains where orographic effects increase MAP to ~1800 mm/yr [76].

A number of paleoecological factors influence the cichlid diversity in Lake Tanganyika. Principle among these factors is the historic variability in lake level. Variation in lake level has been driven primarily by two major forces: tectonism and climate. Below, both mechanisms for lake level change will be summarized chronologically, and lake level lowstands will be identified. In addition, the historic connections between Lake Tanganyika and other water bodies in East Africa will be explored to (1) identify the likely origin of Lake Tanganyika's cichlids and (2) identify possible migratory pathways for Tanganyikan cichlids that colonized Lakes Victoria and Malawi.

#### 2.1.1. Tectonic History of Lake Tanganyika

Lake Tanganyika formed as a series of half-grabens, which are down dropped blocks of land that are bordered by normal faults, within the western arm of the EARS. The geometry of rifting in Tanganyika is highly dependent upon the prerifting basement terrain and the remnants of a preexisting Permian-aged ancient rift [79, 80]. The series of half-grabens are ~160–180 km long by 30–60 km wide [79]. The half-grabens alternate east and west along the length of the lake and are deepest along the border faults and slope upwards towards the opposite shore via a series of faults and folds [79]. They are separated by bathymetric highs known as “high-relief” and “low-relief” accommodation zones forming intra- and interbasinal ridges that separate the lake into three subbasins [81].

The onset of rifting, the evolution of the lake basin, and the early history of Lake Tanganyika are dated relatively imprecisely because there are no direct dates from that time interval. Most of the lake basin's early record has been dated using the reflection seismic-radiocarbon method (RSRM). RSRM estimates ages using sediment thickness estimates derived from reflection-seismic data combined with short-term sedimentation rates calculated from radiocarbon-dated cores. There is some inherent uncertainty in the reflection-seismic estimates of sediment thickness, and RSRM must make the sometimes tenuous assumption that sedimentation rates do not vary through time. Therefore, RSRM age estimates have large uncertainties. The RSRM ages discussed in this paper must be interpreted cautiously until corroborated by direct dating methods. The more recent history of Lake Tanganyika is very well constrained, and the dates for the last ~150 ka can be considered very reliable because they are derived from sediment core data.

Persistence of previous drainage patterns is a common early-stage feature in developing rifts [82]. Based on this analog, it has been suggested that prior to the Miocene until the onset of rifting, the area that would become Lake Tanganyika might have been the site of an ancient river system that drained primarily through a paleo-Congo river system [5, 83]. However, this potential river drainage pattern is uncertain. Rifting probably began ≥9–12 Ma in the central basin and extended northward, then southward [55, 79]. The creation of the Kivu-Ruzizi dome on the north end of modern Lake Tanganyika probably also occurred during this time [18]. The central subbasin infilled first, followed by the north basin, and finally the south basin as the rift opened [13, 55, 84].

Detailed seismic studies of the early history of Lake Tanganyika have been primarily focused on the north basin [13, 55, 84]. Each subbasin in Lake Tanganyika is structurally distinct, and the seismic record from the north basin cannot necessarily be extrapolated to the central and southern subbasins. However, significant tectonic changes and major sequence boundaries documented in the north basin may represent lakewide events. Much of the early tectonic and lake level history presented here is based on data from the north basin and thus must be cautiously interpreted until the events are also documented in the central and southern basins.

The timing of the earliest deposition in the central basin is difficult to resolve [20]. However, there is evidence that during early stages of rifting within the north basin, roughly 7.5 Ma, deposition began as a small, swampy lake formed and then expanded to fill the developing rift [13, 55, 84]. From the onset of rifting until about a million years ago, extension and faulting continued during which time the half-grabens found in each subbasin formed [13, 84]. The end of this initial rifting and depositional phase is marked by a nearly lakewide erosional surface [13, 84].

Following this early period of extremely active tectonism, a second period of geologic activity associated with modification of the existing half-grabens, uplift within the subbasins, renewed volcanic doming, and formation of syn-rift deposits occurred in the northern basin from about one million years ago until ~0.40 Ma [13, 47, 84]. The end of the second period of tectonism and deposition is marked by an erosional surface, which is thought to represent a lowstand event in the northern basin [13]. Subsequently, the basin has been largely inactive with only limited small-scale faulting occurring, allowing the formation of Lake Tanganyika's modern subbasins [13].

#### 2.1.2. Lake Level History of Lake Tanganyika

Lake level has fluctuated dramatically throughout the history of Lake Tanganyika. During the first phase of deposition from approximately seven and a half million to one million years ago, there is evidence for several major unconformities in the northern basin [13, 84, 85]. The timing of these events is difficult to resolve; however, they are most likely related to both tectonic and climatic factors [13]. During the initial phase of tectonism, proto-Lake Tanganyika grew to fill the developing rift basin, and at approximately three and a half million years ago, there is evidence for a dramatic expansion in the size of proto-Lake Tanganyika in the north basin, either as a result of downwarping or due to a transition to a wetter climate across Africa [13]. Following this high stand at about three and a half million years ago, there is a pronounced aridification trend across Africa that is associated with Northern Hemisphere glaciation [39, 48, 52, 53, 63, 64, 86], which likely affected lake level.

The next phase of tectonism began at approximately one million years ago. During this time, lake level in the northern basin was 650–700 m below present lake level (bpll) [13]. The onset of this lowstand is unclear, but it was likely prolonged and may have begun considerably earlier than one million years ago [20, 85]. Based on the modern bathymetry of Lake Tanganyika, a reduction in lake level this large could have split the lake into three hydrologically and biologically isolated basins [83] (Figure 3). However, continued tectonism over the last one million years suggests that the bathymetry of the Lake Tanganyika could have significantly changed during that time. Further, no estimates of lake level in the central and southern subbasins have been made for this time interval. Until new data from the central and southern basin are obtained for this interval, this separation into three subbasins is somewhat speculative.

Following this lowstand, lake level fluctuated dramatically, and the lake significantly contracted in the northern basin several times at ~390–~360, ~290–~260, ~190–~160, ~120–~100, ~40, and 32–14 ka [13, 15, 20, 47, 87–89]. These lowstand events were related to either tectonic factors (~390–~360 and ~190–~160 ka) or major intervals of aridity (~290–~260, ~120–~100, ~40, and 32–14 ka). During the first three lowstand events at ~390–~360, ~290–~260, and ~190–~160 ka, the deepest areas in the lake may have either been separated or have only been connected by emergent, swampy areas [13]. Since 106 ka, Lake Tanganyika has remained a single connected water body, even during significant intervals of aridity [20]. All of these lowstand events, except for the most recent events (younger than ~150 ka), have been well documented mainly in the lake's northern basin, and the exact ages of the events are somewhat uncertain because they were made using RSRM. These RSRM ages for the lake level change events must be corroborated by direct dating methods before they can be considered reliable enough for calibration purposes. Further research in the central and southern basins is also needed to determine whether all of the lowstand events were lake wide, which would indicate a climatic, rather than a tectonic process.

#### 2.1.3. History of Connectivity

Uplift related to rifting processes has caused Lake Tanganyika to have a highly dynamic history of connections to many of the major lakes in East Africa. These variable connections between Lake Tanganyika and the other waters of East Africa have allowed the dispersal of many cichlid lineages, including the haplochromines that seeded the highly diverse species flocks in Lakes Malawi and Victoria [23].

In the Cretaceous and early Cenozoic, prior to the initiation of rifting in the eastern arm of the EARS, the main drainage direction was west to east across the African continent into the proto-Indian Ocean [90]. The uplift of the East African Plateau and the initiation of rifting in the eastern arm of the EAR reversed the drainages west of this propagating rift causing the rivers to flow from east to west [90]. This suggests that as the central basin of Lake Tanganyika formed, it was probably infilled by rivers draining from the east. The most likely source was the proto-Malagarasi River inflow and Lukuga River outflow river system, which may be the only modern river system that also existed prior to the formation of the Tanganyika Rift [47, 78]. Rifting propagated northward from the central basin, and by about seven and a half million years ago, the northern basin had begun to form and was being infilled by a proto-Rusizi River [13]. This represents an early connection between Lake Tanganyika and the Rusizi-Kivu Basin, which was probably also forming at this time [91]. Variable rifting-related uplift has probably led to periodic connections between the Rusizi-Kivu and Tanganyika Basins [91]. These periodic connections may have also allowed for a direction connection between Lakes Kivu and Tanganyika after the formation of Lake Kivu ≥ 2 Ma [91]. The pre-Pleistocene history of this Kivu-Tanganyika connection is poorly understood, however, as result of the general lack of seismic data from Lake Kivu. Today the Rusizi River, which flows from Lake Kivu, is one of Lake Tanganyika's main inlets. Most recently, this connection was likely open ~13–9.5 ka when volcanism in northern Lake Kivu blocked its northern outlet to the Nile [92–94]. This inflow from the Ruisizi River increased lake levels in Tanganyika, causing renewed outflow via the Lukuga River [88]. Following these openings, the Rusizi inlet and Lukuga outlet have closed and reopened multiple times during the Holocene [47, 88, 93, 95]. It is possible that the Tanganyikan cichlids used the connection to Lake Kivu via the proto-Rusizi River to colonize northern bodies of water such as Lake Victoria. Alternatively, the connection between Lake Victoria and Lake Tanganyika may have been through the Malagarasi River, which may have been connected to both Lakes Victoria and Tanganyika during its geologic history.

Lakes Malawi and Tanganyika have a more complex relationship. Today, the two are not connected; however, the Malawian haplochromine cichlids are clearly derived from the Tanganyikan haplochromines [23]. Thus, fish from Lake Tanganyika migrated to Malawi via an unknown riverine connection.

### 2.2. Evolution and Diversification of Tanganyikan Cichlids

Lake Tanganyika contains one of the most diverse fish faunas in the world. Though the exact number of fish species in Lake Tanganyika (or any of the three Great Lakes) is unknown, estimates suggest that Lake Tanganyika supports more than 365 species of fish, at least 115 of which are noncichlids [58, 96]. Depending on how one groups these fish, the cichlids of Lake Tanganyika span either 12 [97] or 16 different tribes [59]. Within these tribes is a remarkable amount of phenotypic diversity in body shape, trophic structures, and behavioral and parental care strategies, making the cichlids of Lake Tanganyika the most phenotypically variable species assemblage in the East African Great Lakes [98]. The unique geological features of the lake, along with the dynamic evolutionary history of its cichlids, serve as an ideal model to study origin of the cichlid diversity [99].

#### 2.2.1. The Origin and Diversification of Lake Tanganyikan Cichlids

Prior to rifting and the formation of any Tanganyikan basin, it has been suggested that an ancient river system that drained west into the Congo River system existed in the location of modern Lake Tanganyika (see above) [83]. This ancient connection between these two bodies of water is further supported by the similarities of fish fauna found in these systems. Eighteen of 24 fish families documented in the Congo are found in tributaries and marshes around the lake. Twelve of the 24 families from the Congo River system occur in littoral and sublittoral zones of the lake. Seven Congolese families are found in the benthic and four in the pelagic zone of Lake Tanganyika [78]. However, recent immigration of these families into this Lake Tanganyika cannot be ruled out.

Among the cichlids, it is clear that the Tanganyikan radiation is nested within cichlids endemic to the Congo River system [100]. According to Schwarzer et al. [100, 101], the East African cichlid radiation is a sister group to a clade containing the substrate brooding genus *Steatocranus *that is common in the Congo River system. Together with the several species of tilapia ([100], clade “AII”), the genus *Steatocranus *and the cichlids of Lakes Tanganyika, Malawi, and Victoria form the Austrotilapiini. The Austrotilapiini are further nested within the larger Haplotilapiini, which itself is nested within a collection of taxa that are widespread across the Congo River system and West Africa. The findings of Schwarzer et al. [100] are largely consistent with those of Farias et al. [74], both of which provide clear support for the Congolese origin of the cichlids of Lake Tanganyika.

The relationship between Tanganyikan and Congolese cichlids, however, is far from simple [24] (Figure 4). Poll [97] originally identified 12 polyphyletic cichlid tribes in Lake Tanganyika and concluded that the lake had been colonized multiple independent times. Since then, a number of molecular phylogenetic studies, summarized by Koblmüller et al. [25], support the multiple invasion hypothesis, and the 12 tribes that originated during the primary lacustrine radiation have been identified: the Haplochromini (including the Tropheini and the hyperdiverse haplochromines of Lakes Victoria and Malawi), the “new tribe” consisting of *Ctenochromis* species, the Cyphotilapiini, the Benthochromini, the Limnochromini, the Perissodini, the Cyprichromini, the Ectodini, the Lamprologini, the Eretmodini, the Orthochromini, and the Bathybatini. After the initial invasion of the lake, many of these lineages experienced a secondary radiation, which formed the modern cichlid diversity found in Lake Tanganyika [99]. Following these radiations, species from several of the radiating lineages recolonized the rivers surrounding Lake Tanganyika [24, 75]. The lineages that secondarily invaded the surrounding rivers include two species rich tribes, the Lamprologini and Haplochromini, and one lineage currently found exclusively in rivers, the Orthochromini. Two additional lineages, the Tilapiini and the Tylochromini, recently invaded the lake [102, 103].

The diversification of the Haplochromini demonstrates the complex patterns of dispersal between the cichlids of Lake Tanganyika and its surrounding rivers. The common ancestor to the haplochromines evolved within a larger, lacustrine cichlid diversification in Lake Tanganyika. These haplochromines then colonized the rivers in the surrounding catchment of Lake Tanganyika. These generalized riverine haplochromines then secondarily invaded the lacustrine habitats in Lakes Tanganyika, Malawi, and Victoria. In each lake, the haplochromines (the “modern” haplochromines) then experienced a remarkable radiation [23]. Thus Lake Tanganyika is not only a sink for ancient African cichlid lineages, but also a source of recent cichlid diversity in East Africa.

In contrast to a predominately intralacustrine radiation, Lake Tanganyika could have been colonized by a larger number of more diverse taxa [75]. When the molecular clock is calibrated to the breakup of Gondwana, molecular clock estimates of divergence times suggest that nearly all Tanganyikan lineages began to diverge prior to the estimated onset of deep lake conditions [75]. In this model, the divergence of Lake Tanganyika's cichlid fishes did not occur in Lake Tanganyika, but rather these lineages began to diversify in the surrounding rivers prior to the formation of the lake. Though novel, this much older estimate of the diversification of Lake Tanganyikan cichlids conflicts with long-held assumptions concerning the habitats suitable for cichlid radiations, the evolution of resource partitioning, and the biogeographic patterns of species distributions [25]. In addition, it is well known that estimating recent diversification events with ancient calibration points may produce unreliable age estimates with large variances [104].

#### 2.2.2. Ages of the Lake Tanganyikan Cichlid Radiations

To resolve these alternative hypotheses, an accurate estimate of the divergence time for Lake Tanganyika's cichlids is needed. Unfortunately, different calibration methods yield highly incongruent estimates. By calibrating the molecular clock to recent geologic events (e.g., the formation of Lake Malawi and the inundation of the Lukaga valley), Salzburger et al. [23] suggested that Lake Tanganyika's cichlids evolved since the formation of the lake basin 6–12 Ma and that the species-rich haplochromines originated approximately 2.4 Ma. While this estimate is widely accepted within the cichlid community, this calibration method relies on assumptions that are still debated within the geologic literature. For example, this calibration method ignores the fact that the timing of Lake Malawi's formation is poorly constrained.

Genner et al. [75] generated age estimates using two calibration methods. One method relied on the cichlid fossil record, while the other relied on the breakup of Gondwana. The cichlid fossil record calibration suggests that Lake Tanganyika's cichlids began diversifying coincident with deep lake conditions (6–12 Ma), a finding that is consistent with previous estimates [23]. Genner et al. [75] favor instead the Gondwana calibration. This calibration suggests that the diversification of Lake Tanganyika's cichlids had begun prior to the creation of Lake Tanganyika's basin, possibly in a now extinct paleolake. However, there is no geologic evidence to support this paleolake hypothesis. Genner et al.'s [75] conclusions were supported by estimates produced by Schwarzer et al. [100] who used the fossil record of *Oreochromis lorenzoi *(5.98 Ma), the divergence of *Tylochromis, *and the remaining East African cichlids (53–89 Ma) as calibration points. However, as was noted by Koblmüller et al. [25], Genner et al.'s [75] Gondwana calibration lacks constraints on the more shallow bifurcations which may lead to the incorrect assignment of divergence times. Koblmüller et al. [105] produced models calibrated to a number of geologic points including the occurrence of deep water conditions in the Great Lakes and Genner et al.'s [75] Gondwana calibration. From this analysis, Koblmüller et al. [105] conclude that the most parsimonious age estimate for the divergence of Lake Tanganyika's cichlids is ~6 Ma with the most recent common ancestor of the haplochromines occurring 5.3–4.4 Ma [105].

It is worth noting, however, that these divergence times are highly dependent on the estimated timing of geologic events that have large uncertainties. Estimating the time since divergence in many cichlid lineages is further complicated by the age of the events used to calibrate the molecular clock. Many of cichlid diversification events occurred relatively recently, while the events used to calibrate the molecular clock are comparatively old (e.g., the breakup of supercontinents, the formation of lake basins) [25, 104]. Thus, the continued analysis of the geologic history of this dynamic region is needed to accurately quantify the divergence times of the East African cichlids [106]. It is clear that the divergence times of Lake Tanganyika's cichlid and the haplochromines have yet to be resolved.

Incomplete taxon sampling is another major limitation of the current dating estimates [105]. In most studies, one or several of the major lineages are not included in the phylogenetic reconstruction. Further, based on recent publications, not all of haplochromines lineages in the region have been identified [26]. Dating estimates are further limited by the lack of good calibration points. Estimates from molecular clocks become more reliable when multiple geological and fossil calibration points are used [105, 107, 108] in combination with reliable rates of sequence evolution [109], estimates of sequence saturation [110], and a posteriori evaluation of estimated divergence times [108, 110]. Few of these requirements have been satisfied in previous analyses, and the necessary data are just now becoming available [106]. Thus, caution is necessary when considering the dates provided here.

#### 2.2.3. Impact of Lake Level Fluctuations

The dynamic geological history and variable paleoclimate of Lake Tanganyika have shaped the cichlid diversity in this lake. The effect of these factors can be seen in three major areas: the maintenance of ancient cichlid lineages, the isolation of populations, and the admixture of previously isolated populations.

East Africa has experienced multiple periods of extreme aridity during the Pleistocene. For cichlid lineages to have persisted through such events, water sources must have remained available. Owing to the great depth of the lake, even during periods of extreme aridity [13, 20], Lake Tanganyika would have been a refugium for ancient cichlid lineages, which is reflected in age estimates of the diversification of Tanganyikan cichlids [23, 75].

Though these periods of aridity apparently did not extirpate the seeding lineages in Tanganyika, the resulting low lake levels likely had a significant impact on the distribution of genetic variation within and among these lineages. For example, an analysis by Sturmbauer et al. [111] of mtDNA regions from several *Tropheus *populations identified 6 phylo-geographically unique haplotype clusters in three regions of the lake: the northern basin, the central basin, and the southern basin. Remarkably, in the central and southern parts of the lake, individuals from one side of the lake had mitochondrial haplotype identical to those found at the opposite shoreline. It is possible that during one of the major regressive events, Lake Tanganyika was separated into three near-distinct lakes (Figure 3), and populations that are currently separated by deep water were connected through the shorelines of these three separate lakes. Alternatively, during times of lake level fall, water levels at topographic highs that separate the subbasins may have become shallow enough to allow species to migrate from one side of the lake to the other. Given that there are discrete haplotypes clusters for each of the three subbasins and that their mitochondrial haplotype are shared by individuals on opposite sides of the lake in each subbasin, the separation into three near-distinct lakes seems to be the more likely scenario.

While the cross-lake affinities of mitochondrial haplotypes reflect the impact of major regressive events on the distribution of genetic variation in Lake Tanganyika's cichlids, the interaction of historic hydrology and bathymetry can have a more subtle influence. Examining the genetic diversity in a collection of *Tropheus moorii *populations, Koblmüller et al. [112] detected the effect that changes in lake level have had on the genetic diversity over extremely limited geographical distances. In this study, two distinct collections of populations were identified. One collection was located on the steeply sloping shores of the eastern side of the Chituta Bay, while the other was located on the more gently sloping shores west of the bay. Within the eastern populations, three distinct populations which corresponded to geographic locations were identified. In contrast, the western populations show greater degree of admixture. The authors conclude that this pattern is consistent with the horizontal displacement of the western shore populations as the lake regressed, causing those populations to admix as the available habitat shrank. The eastern populations migrated vertically along the steeply sloping habitat, were not forced into secondary contact and retained their accumulated genetic differences. In both the eastern and western populations, the authors detect the signature of population expansion that coincided with the end of the Last Glacial Maximum. Population expansion was greater in the western populations likely as a consequence of this area having relatively greater area of available habitat with a rise in lake level. The authors conclude that rapid, dramatic, and relatively recent climatic changes in East Africa drive both population divergence and population admixture.

## 3. Lake Malawi

### 3.1. Paleoclimate and Geologic History of Lake Malawi

At 700 m deep, 580 km long, and 30–80 km wide, Lake Malawi is one of the largest lakes in the world. Rifting in the Malawi Rift began during the Late Miocene, probably no less than ~8.5 Ma, and propagated from north to south resulting in three drainage basins [6, 7, 56, 85, 91, 113–116]. The two northernmost drainage basins are deeper and steeper sided, while the southern basin is shallower with a muddy bottom [19, 116]. The age of the formation of Lake Malawi is very uncertain. Geologic evidence from deposits surrounding the lake suggests that a deep lake may have first existed between ~4.5 and 8 Ma [6, 7, 56, 57, 114, 115]; however, it is possible that a lake was present even earlier during the earliest phases of rifting, 8–12 Ma [57, 117]. Since formation, the lake has undergone dramatic fluctuations in lake level during its history [15, 17, 85, 118].

The climate of Lake Malawi is strongly influenced by the seasonal migration of the ITCZ producing a wet-dry monsoonal cycle with the wet season extending from December to April. Annual precipitation is seasonal and ranges from <800 mm/yr in the south to >2400 mm/yr in the north [119]. The lake is close to the southern extent of the modern ITCZ path (Figure 2), and thus changes in the position of the ITCZ and its intensity considerably affect dry season length and have been linked to periods of aridity and drops in lake level during the Pleistocene [15, 17, 32].

Lake Malawi is permanently stratified with a chemocline depth of ~250 m [120], and today the lake is hydrologically open. Several large drainage systems enter the lake across different structural settings in the three drainage basins [121], and the sole outlet is the Shire River. Although outflow to the Shire is continuous, more than 90% of water loss in the lake is due to evaporation [119, 122, 123]. Because precipitation is seasonal and evaporation is the main contributor to water loss, lake level seasonally fluctuates up to a few meters. Variability in lake level has caused disruption of the outflow during historical times [72] and frequently throughout the geologic history of the lake. Considerable reductions in lake level during the lake's geologic history have caused the outlet to be disrupted, and the lake has become closed and more saline [18, 22].

The geologic and paleoclimatic history of Lake Malawi, which has influenced the connectivity of Lake Malawi to the surrounding waters and generated highly variable lake levels, has played an important role in the evolution of its endemic species. Below we review the geologic and paleoclimatic history of Lake Malawi and discuss their influences on the divergence of its haplochromine flock.

#### 3.1.1. Tectonic History of Lake Malawi

The Malawi Rift is located at the southern end of the western arm of East Africa rift between 9°and 14°S, and almost two-thirds of the rift is filled by Lake Malawi. The rift zone is comprised of four alternating asymmetrical half-grabens and several smaller basins, resulting in three main structural and drainage basins [114, 116]. Each half-graben is bounded by a steep border fault with high rift mountains (>1500 m above lake level) along the lake shore on one side and a shallower flexural or shoaling margin on the opposite side [121]. The border faults link across “transfer zones” [85] which strongly influence drainage and deposition patterns in each of the basins [124, 125]. The long-lived half-graben basins and associated deep subsidence have resulted in the development of a long-lived lake basin [95, 116]. As a result, the lake is underlain by >4000 m of lacustrine sediment that thins from the far north to the south, indicating that rifting has propagated from north to south [2, 113–116]. The northern and central basins, which are up to ~700 m deep, are each ~150 km long and are characterized by very steep offshore slopes at the border faults [116, 125]. In these basins, sediment is primarily transported downslope within channels and canyons and out onto well-developed fan complexes [125, 126]. The offshore slopes in the shallower southern basin (maximum depth = 450 m) are less steep than the northern and central basins, and the basin is primarily covered by fine-grained, muddy sediments [125, 126].

The exact timing of the onset of rifting is unknown; however, the earliest sediments that are associated with Cenozoic rifting in the Rungwe volcanic province, which borders the Malawi Rift to the north, are associated with welded tuffs dated to 8.6 Ma [7, 56]. The sediments are not directly correlative with sediments in the subsiding Malawi Rift; thus, 8.6 Ma is a minimum age for the onset of rifting. Age models based on sedimentation rates suggest that rifting commenced between 8 and 12 Ma [117]. The earliest interval of rifting and subsidence in the Malawi Rift probably occurred contemporaneously with two pulses of volcanism in the Rungwe volcanic province between 8.6 and 1.7 Ma during which most faulting occurred parallel or subparallel to the bounding faults [7, 56, 57, 117]. This was then followed by an ~1 Ma interval of quiescence until the latest phase of rifting beginning at ~500–400 ka when there was a shift in rifting style to oblique rifting and strike-slip deformation [117, 127].

#### 3.1.2. Lake Level History of Lake Malawi

The early history of Lake Malawi is somewhat difficult to resolve. Radiometric dates from lavas and tuffs surrounding Lake Malawi [7, 56] and sedimentological evidence suggest that a small, shallow lake may have periodically existed after the onset of rifting [57]. Lacustrine deposits, structural evidence, and an increase in the rate of subsidence of the lake floor between 4.5 and 1.6 Ma suggest that a deep lake formed by ~4.5 Ma, if not earlier [57, 128, 129].

Sedimentation in the Malawi Rift occurred contemporaneously with two of the early pulses of the Rungwe Volcanic province to the north of the lake between 8.6 and 1.7 Ma [7, 56, 57]. During this period of volcanism, rifting, and deposition, there is evidence for multiple depositional hiatuses that likely correlate with significant reductions in lake level [57]. The age of the early hiatuses is poorly constrained. Two of them may be contemporaneous with onshore unconformities that have been dated to 2.3 and 1.6 Ma [56]; however, it is uncertain to which offshore unconformity the lake events correlate. This onshore evidence indicates a pronounced unconformity from 1.6 to 1.0 Ma during which time Lake Malawi was probably significantly reduced in size and possibly even completely desiccated [56, 57, 127] (Figure 3). Preliminary evidence from drill core records also suggests that Lake Malawi was a significantly reduced, saline lake at ~1.2 Ma [22]. Following this lowstand, lake level rose towards deeper lake conditions [22]; however, the history of lake level change is poorly resolved from 1.2 to ~0.15 Ma.

Between ~150 and 60 ka, there were dramatic fluctuations in lake level [15, 17–19]. During this time period, there were two intervals of pronounced lake level drops up to 550 m bpll: one from 135 to 124 ka and the other from 117 to 85 ka [15, 17, 19]. These two megadrought events would have severely restricted Lake Malawi, and lake level may have been reduced to as little as 2% of modern lake levels [19]. Between the two megadroughts was an interval of relatively high lake levels where the lake was stratified and the bottom water was anoxic [18, 19]. Beginning at ~60 ka, the lake rose to much higher levels, and changes in lake level were much less dramatic than during the preceding 90 ka. There have been modest fluctuations in lake level (100 m or less) since 60 ka, including during the LGM [19]. However, in general lake conditions during the last 60 ka have been relatively stable and similar to those at present.

### 3.2. Evolution and Diversification of Malawian Cichlids

In many respects, the origin and diversification of Lake Malawi's cichlid fish is the least complex of the three Great Lake radiations. Lake Malawi contains both tilapiine and haplochromine cichlids. The tilapia are represented by two distantly related lineages [102]: *Tilapia rendalli,* a substrate spawning species common throughout the region, and an endemic species flock, the chambo, containing three species (*Oreochromis karongae, Oreochromis lidole,* and *Oreochromis squamipinnis*) [130]. Given the paucity of endemic tilapiine species, this section will focus on the more diverse haplochromine lineage.

#### 3.2.1. Origin and Diversification of Lake Malawi's Haplochromine Cichlids

With over 700 endemic species [58] most, if not all of which appear to have descended from a single common ancestor [131], the haplochromine cichlids of Lake Malawi are the largest monophyletic species flock of cichlid fishes. This species flock is nested within the Lake Tanganyikan haplochromine group and is sister to the clade containing the haplochromine cichlids of the Lake Victoria superflock [23].

The age of Lake Malawi's species flock, like the ages of other East African cichlid flocks, is debated. Sturmbauer et al. [111] calibrated the age of Lake Malawi's cichlids to the geologic history of the lake [57] and estimated the divergence of Lake Malawi's cichlids at 0.93–1.64 Ma. In contrast, Genner et al. [75] using two calibration methods (see above) suggested that Lake Malawi's cichlid flock originated either 4.6 Ma (Gondwanan calibration) or 2.4 Ma (fossil calibration). Genner et al.'s [75] estimate suggests that Lake Malawi's cichlids began to diversify prior to deep water conditions in the lake and/or persisted through multiple megadroughts that either desiccated the lake or dramatically altered the water chemistry thereby making the lake uninhabitable. Genner et al.'s [75] estimates were not supported by the work of Koblmüller and colleagues [25]. In Koblmüller et al.'s [25] analysis, the estimated age of the Lake Malawi's cichlids ranged between 0.72 and 1.80 Ma for five of the seven calibration methods used. Though the estimated age of Lake Malawi's cichlid flock is not known, an abundance of data suggests that this flock originated ~1 Ma. If or how Lake Malawi's haplochromine cichlids persisted through the megadroughts of 135 ka and 117 ka is unknown.

Assuming an origination age of ~1 Ma for the Malawi cichlid flock, a riverine generalist similar to *Astatotilapia calliptera *or *Astatotilapia* *bloyeti *[105] migrated from Lake Tanganyika to Lake Malawi during that time [23] (Figure 4). The cichlids of Lake Malawi then diverged into two large clades plus several oligotypic lineages. The two large clades, each containing ~250–300 species, are reciprocally monophyletic and consist of the rock-dwelling cichlids, or mbuna, and the sand-dwelling cichlids [132]. Genner et al. [75] suggest that the mbuna emerged 0.486 Ma (Gondwanan) or 0.313 Ma (fossil), while the more diverse sand-dwelling cichlids emerged 1.447 Ma (Gondwanan) or 0.855 Ma (fossil). Given the large variances of these estimates and lack of multiple calibration points [25], the reliability of these divergence estimates is unknown. Future research utilizing a broad sampling of taxa and multiple calibration points is needed to accurately estimate the ages of these highly diverse clades.

#### 3.2.2. Impact of Lake level Fluctuations

Regressive events probably played an important role in shaping the evolutionary history of Lake Malawi cichlids. For example, Genner and Turner [133] recently discovered that one of Lake Malawi's most diverse clades evolved in response to a major regression event. Between ~75 and 135 ka, lake level dropped as much as 580 m bpll [15, 17]. As a consequence, the shallow benthic habitats of southern Lake Malawi completely desiccated, thereby reducing the proportion of shallow rock and sand habitats relative to deep benthic and pelagic habitats. In addition, this lowstand facilitated the hybridization of two diverging lineages: the rock dwellers and the sand dwellers. As a consequence of this event, this new hybrid lineage rapidly adapted to the now plentiful deep benthic habitats and gave rise to as many as one-third of all the species in Lake Malawi's haplochromine radiation.

During the following transgressive period which brought the lake to its current level, the littoral areas north and south of the central basin were reinundated. The newly emerging habitats provided the opportunity for expansion and diversification of many species, which is reflected in both the patterns of genetic [134] and species diversity [135]. This period that reestablished littoral habitats and permitted the rapid expansion of populations is likely synchronized with similar phenomenon in other East African lakes [111, 136].

Within historical times, Lake Malawi has experienced meaningful but less dramatic regressive/transgressive events. For example, Owen et al. [72] found that much of southern Lake Malawi was exposed land as recently as 300 years ago. Given the large number of species endemic to this area [135, 137, 138], a regressive event of this magnitude would suggest an exceptionally rapid diversification of southern Lake Malawi endemics. These rapid and recent regressive/transgressive events are believed to have disrupted and permitted gene flow between mbuna populations and thereby contributed to the high cichlid diversity in Lake Malawi [139].

## 4. Lake Victoria

### 4.1. Paleoclimate and Geologic History of the Lake Victoria Region

Lake Victoria is the largest freshwater lake in the tropics by surface area (68,800 km^2^) and the second largest in the world. The lake spans the equator in between the western and eastern branches of the EARS (Figure 1), giving it a rectangular-shaped coastline. The timing of the formation of Lake Victoria is uncertain. The lake has been suggested to have formed between 1.6 and ~0.40 Ma due to backponding associated with the damming of westward flowing rivers by the uplifting of the western arm of the EARS [7–12]. Unlike the other African Great Lakes, Lake Victoria is not within a rift basin, and as a result, it is relatively shallow with a maximum depth of less than 100 m.

Modern climate in the Lake Victoria region is primarily controlled by the ITCZ, which crosses Lake Victoria twice a year in March (long rains) and again in October (short rains) [140]. The mean annual precipitation of the Lake Victoria region is ~1600 mm/yr [141]. The lake is monomictic, and mixing by the trade winds occurs during the dry season between May and August [142]. However, in modern times, the lake's water column has become more stable, so that as much as 40% of the lake's bottom waters are anoxic [143].

Today, the lake is hydrologically open with its major inlets being the Kagera and Katonga Rivers in the west. The primary outlet is the Victoria Nile at the northern end of lake. As much as 90% of water loss is from evaporation and 80% of the input is from direct precipitation on the lake surface [141, 144]. Because evaporation is consistent, whereas precipitation in the Lake Victoria region is variable, changes in precipitation have profound impacts on lake level [145]. Due to its shallow depth (<100 m) and strong dependence on precipitation to maintain lake level, Lake Victoria has desiccated completely multiple times, probably in response to increased aridity [10, 14, 16, 21, 70].

The geologic and paleoclimatic history of Lake Victoria is considerably different than that of Lakes Tanganyika and Malawi. Principal among these differences is the depths of the lakes and the influence of arid intervals on the lake's persistence. Lake Victoria is a relatively young, shallow lake that completely desiccated ~15 ka. Despite this event, the cichlids of Lake Victoria are species-rich and widely distributed outside the lake basin [27, 28]. Below we describe the geologic and paleoclimatic history of Lake Victoria and its surrounding waters and discuss how the geologic and paleoclimatic history has influenced the extensive radiation of the Lake Victoria cichlid superflock.

#### 4.1.1. Evolution of Lake Victoria Basin

Prior to the onset of rifting in the Miocene, the Lake Victoria region drained from east to west. Rifting in the western branch of EARS during the late Miocene and the Pliocene probably created an NE-SW-oriented basin that began to capture some of the tributaries feeding the Congo River [12, 146]. Eventually rifting in the western EARS completely truncated this network during the Pleistocene, causing the rivers to flow eastward [7]. This eastward flow from the western branch of the EARS, coupled with the westward flow of rivers draining the western flank of the eastern EARS [147], formed Lake Victoria as the low-relief areas between the two arms of the EARS filled with water. The timing of this formation is poorly constrained, and the maximum estimate for the timing of formation is ~1.6 Ma [9–11], but because the lake formed by backponding between the two arms of the EARS, it is possible that Lake Victoria, or a “proto-Lake Victoria,” formed earlier than 1.6 Ma.

After formation of the lake, rifting continued to tilt the basin eastward, moving the center of lake 50 km to the east and exposing mid- to late-Pleistocene lacustrine sediments west of the lake [11, 143]. These sediments are exposed in the Kagera River Valley 100 km to the west and 130 m above present lake level. Doornkamp and Temple [11] used these sediments to suggest that Lake Victoria was younger than 0.8 Ma. Mid-Pleistocene lacustrine sediments identified in Kenya near the Kavirondo Gulf have been used to suggest that Lake Victoria may be as old as 1.6 Ma [9]. However, it is important to note that these dates (0.8 Ma and 1.6 Ma) are very imprecise and poorly constrained. Additional work is needed to better constrain the age of these lacustrine sediments surrounding modern Lake Victoria. RSRM estimates for the 60 m thick package of sediment in Lake Victoria suggest at least 0.4 Ma of deposition [10]. However, there are multiple hiatuses in the succession, and their durations are not possible to estimate, indicating that 0.4 Ma is a minimum estimate for the formation of the lake [10].

The original outflow of Lake Victoria was probably to the west directly into Lake Albert [148]. Uplift associated with continued rifting of the EARS likely blocked this connection and established the modern outflow through Lake Kyoga by ~35–25 ka [148]. The first connection of Lake Victoria to the White Nile may have been as early as ~0.4 Ma [149]. The timing of the modern connection of Lake Victoria to the White Nile via the Victoria Nile is uncertain though it has probably occurred in the last 13 ka [8].

#### 4.1.2. Paleoclimate and Paleoenvironment

Lake Victoria is extremely dependent on precipitation because as much as 80% of water input is from direct precipitation on the lake surface [118, 141, 144]. Seismic data indicates that lake level has fluctuated significantly during the Pleistocene and Holocene and that there were multiple intervals when the lake completely desiccated [10]. Coring of the uppermost sediments provides evidence for the most recent desiccation events. Near the base of the core is a 16-17 ka paleosol that represents drying at the end of the LGM [10, 14, 16]. This paleosol has shrink-swell features that identify it as a paleo-Vertisol [10]. In order to form a Vertisol, the soil must be completely desiccated for at least one month per year [150]. Seismic data indicate that the paleo-Vertisol can be traced continuously across the entire lake basin [10, 14, 16, 143]. The bathymetry of Lake Victoria does not allow for the formation of separate basins where smaller lakes could have persisted [10, 14, 16, 143], leaving no refugium for cichlids. Following this desiccation event during the LGM, the lake dried up again between 14 and 15 ka [14, 16, 21]. Thus, Lake Victoria was completely desiccated for at least two intervals from the LGM to ~14 ka, and it is highly unlikely that the lake could have supported any cichlid populations during these events. Following these desiccation events, the lake probably filled relatively quickly [21].

In Lake Albert, two paleosols have been identified between 18 ka and 12.5 ka, indicating that Lake Albert probably also desiccated at least twice since the LGM [151]. Other evidence from the Burundi Highlands and from the Congo River Basin also suggests that the late Pleistocene in equatorial East Africa was arid [152, 153]. This evidence for aridity coupled with the roughly contemporaneous paleosol horizons in Lakes Victoria and Albert suggests that many of the lakes in which the Lake Victoria superflock currently persists (e.g., Lakes Victoria, Albert, George, and Kyoga) were probably completely dry for at least some period of time during the LGM and the subsequent arid interval during the latest Pleistocene.

Across Africa, the early- to mid-Holocene was generally much wetter [86], and by ~12 to 13 ka lake levels in Lake Victoria and other surrounding lakes began to fill to their current level [21, 143, 154]. Throughout the Holocene, Lake Victoria experienced changes in lake levels, but no other complete desiccations [21]. During the last 4 ka, climate has become more seasonal, and precipitation has decreased, which has likely caused lake level to decrease such that Lake Nabugabo separated from Lake Victoria [21, 155–157].

### 4.2. Evolution and Diversification of the Lake Victoria Superflock

The evolutionary history of the cichlids of Lake Victoria cannot be fully understood without a broader discussion of the greater Lake Victoria species flock. While Lake Victoria supports at least 150 endemic species of cichlids, this diversification is only a fraction of cichlids belonging to the Lake Victoria superflock (LVSF) [27]. The superflock consists of over 600 species of haplochromine cichlids spread across nearby lakes such as Lakes Albert, Edward, George, Kyoga, Kivu, and the rivers of the region [27], in addition to more distant locations such as the more southern Lake Rukwa and its drainage [27], Lake Turkana [105], smaller North-Eastern Tanzanian lakes [26], and water bodies as far north as Egypt, Tunisia, and Israel [29, 30, 105]. Thus, the LVSF has a geographic distribution far larger than those of Lakes Tanganyika and Malawi [28, 158].

In order to understand the complex relationships of fish in this superflock, multiple phylogenetic, biogeographic, and population genetic studies have been performed. These have revealed a complex phylogeographic pattern, which reflects the influence of past geological and climatic events on the colonization of new habitats [26, 28, 105]. Below we examine these patterns with special attention given to identifying the geographic origin of the superflock, its age, and how its members persisted through recent periods of extreme aridity in East Africa.

#### 4.2.1. Age and Origin of the Lake Victoria Superflock

Molecular phylogenetic evidence indicates that the LVSF predates the most recent complete desiccation event at ~14-15 ka. The LVSF appears to have emerged at about 200 ka [159] with major diversification of lineages between 98 and 132 ka [28]. Similar patterns in which genetic lineages predate the refilling of the Lake Victoria basin were found in cyprinid fish [160], catfish [161], and snails [162, 163]. However, all of these estimated ages rely on the estimated timing of geological events which themselves may be revised through future research (see above).

On the basis of these estimates of lineage divergence, several authors suggested that the lake never completely desiccated [164, 165]. Yet, the geological evidence is unambiguous. Lake Victoria and its surrounding waters were completely dry at least once, and possibly twice, between ~14 and 20 ka [10, 14, 16, 143, 151]. This conclusion is consistent with phylogenetic analysis of the superflock. Based on estimates of speciation rates for all cichlid lineages in the Lake Victoria region, Seehausen [166] did not reject the Pleistocene desiccation event and concluded that the lake was colonized from a source outside of the basin rather than persisting in small refugia within the basin itself. Hence, the major diversification of the modern cichlid superflock in the Lake Victoria region coincides with the Holocene refilling of the lake when large areas of habitat became available again. This conclusion is supported by the apparent severe bottleneck [136] and subsequent range expansion [111] that occurred in this lineage. Together these studies support the conclusion that the present cichlid diversity endemic to Lake Victoria must be the result of recent colonization followed by intralacustrine speciation.

The rapid diversification of the LVSF has been attributed by some [167] to the formation of a hybrid clade leading to morphological novelty. Such hybridization events at the base of highly diverse clades have been documented in Lakes Tanganyika [168] and Malawi [60]. A similar event may have influenced the origin of Lake Victoria's cichlids [169].

To find the source of the lineages that colonized the Lake Victoria region, multiple phylogenetic and population genetic studies have been performed. These studies identified several potential colonization sources, including the Kagera and Katonga Rivers [170] and the Congo [171]. Another possible source for the ancestral lineages could have been paleo-Lake Obweruka that formed 8 Ma but desiccated during the late Pliocene [1]. Paleo-Lake Obweruka matched Lake Tanganyika in size and depth and hence provided a large area of habitat [1]. Such paleo-lakes have been implicated in the diversification of related cichlid taxa. Joyce et al. [172], for example, showed that much of the riverine cichlid diversity in southern Africa can be traced back to paleo-Lake Makgadikgadi, where the group radiated before the lake desiccated and the species dispersed into the surrounding waters. However, geological estimates of the desiccation of paleo-Lake Obweruka indicate that it likely did not play a role in seeding the modern constituents of the LVSF because it disappeared before the formation of modern Lake Victoria. Yet another hypothesis suggests that the LVSF arose out of a lineage that persisted in refugia in Tanzania. The lakes in the Eastern Arc region of Tanzania did not desiccate during the Pleistocene and have been suggested to have served as refugia for the Lake Victoria species flock [25, 173]. This, however, seems unlikely. Hermann et al. [26] demonstrated that the cichlids found in that region represent an ancient lineage which is not closely related to the LVSF. There now seems to be general agreement that the Lake Victoria superflock arose more recently in the much smaller Lake Kivu [28, 136] (Figure 4).

#### 4.2.2. The “Out of Kivu Hypothesis”

Lake Kivu harbors 15 endemic haplochromine species in addition to three tilapiine species one of which is native (*Oreochromis niloticus*) while the remaining two (*Oreochromis macrochir, Tilapia rendalli*) were introduced [174]). Among the haplochromine species, two phylogenetically distinct lineages can be distinguished both genetically and phenotypically [175]. Interestingly, these two groups correspond to the two lineages of haplochromines found in Lake Victoria [175]. Furthermore, phylogenetic and population genetic evidence clearly indicates that the ancestors of the superflock are derived from Lake Kivu's haplochromines [28, 136]. Molecular clock estimates suggest that the split between the Lake Victoria and Kivu lineages occurred less than 41.5–30.5 ka. This estimate roughly coincides with the eruptions of Virunga volcanoes (25–11 ka), which disrupted the connection between Kivu and the northern lakes in the Lake Victoria region (Lakes Albert, Edward, George, and Kyoga) [28]. However, a causative relationship between the split of Lake Kivu's and Lake Victoria's cichlids and the eruptions of the Virunga volcanoes is speculative. The apparent similarities in the timing of these events depends on a geologic calibration point, the origin of lacustrine conditions in Lake Malawi [57], which is relatively poorly constrained.

Lake Kivu may be an important, though not ultimate, source of cichlid diversity. While at least two lineages from Lake Kivu have invaded the Lake Victoria region and diversified, it appears that a third lineage left Lake Kivu earlier and seeded a smaller radiation in North Eastern Tanzania [26]. The lineage might have been separated from the western cichlids during the formation of the Kenyan-Tanzanian rift system formation [26].

#### 4.2.3. Relationships within the LVSF and between the Other Lakes

While most researchers agree on the postdesiccation colonization and diversification of Lake Victoria's endemic cichlids, the number of invading lineages appears to be less clear. Nagl et al. [27] suggested that the lake has been invaded at least twice, which is consistent with the results of Verheyen et al. [28]. Additionally, this study found evidence that Lakes Albert, George, and Edward have been seeded at least four times. It appears, however, that only one of the four invading lineages radiated extensively, while the others are rare relicts. Although the number of colonizers remains unclear, some phylogenetic patterns can be found within the LVSF.

Nagl et al. [27] found seven haplogroups within the Lake Victoria superflock. Two lineages (II, IV) are found only at and around Lake Rukwa. A third lineage is restricted to Lake Manyara and Tanzanian rivers (VI). A fourth lineage is found in these same rivers (III), while a fifth lineage is restricted to the Malagarasi River east of Lake Tanganyika (I). A sixth lineage (VII) is found in the Malagarasi River, the Kazinga Channel, and Lake George. However, all species endemic to Lake Victoria and its surrounding lakes and rivers fell into a single haplogroup (V). Within this haplogroup V, four subgroups were distinguished: one is endemic to Lake Victoria (VD), one is found in the rivers close to Lake Rukwa (VA), and the other two lineages have a wider distribution within the Lake Victoria region and can be found in Lakes Victoria, Albert, Edward, and George and adjacent rivers (VB, VC).

While these relationships within the lake and the region are fairly complex, the phylogeography of the superflock becomes even more complicated when one considers members of the LVSF that occur in water bodies far from Lake Victoria. Members of the LVSF have been found as far south as Lake Rukwa [27], in the North Eastern Lake Turkana [25], and as far North as Egypt and Israel [25, 29, 30]. These distributional patterns are interesting from a biogeographical as well as from a paleogeographical standpoint since they inform on past connectivity and colonization events. For example, the presence of members of the superflock in Lake Rukwa has been explained by a series of river capture events that might have enabled the colonization of Lake Rukwa from the Lake Victoria region [25]. Lake Turkana was probably colonized fairly recently in the early Holocene, when Turkana spilled over into the Nilotic system and a connection between the Turkana and the Nile was established [176]. This is supported by a fairly young age of the Lake Turkana endemic species *H.  rudolfianus *which groups with the LV species flock [25]. Northern African locations in turn were likely colonized ~11 ka via the Nile [25].

## 5. Biogeographic Implications

The cyclical periods of aridity/humidity and the resulting contraction, diversification, and expansion of species resemble the classic biogeographic theory formulated by Bush [177] to explain Amazonian diversity [177]. In Bush's [177] diversity-instability hypothesis, species become fragmented due to climatic changes associated with glacial/postglacial environmental conditions [177]. While fragmented, these species undergo allopatric speciation. Repeated bouts of climatic change through the Pleistocene would act as species pumps that increase the species diversity in the tropics.

It is clear that similar climatic cycles have influenced the diversification of East African cichlids [60, 111, 112, 134, 136, 166]. In East Africa, periods of humidity facilitated the dispersal and fragmentation of species [23, 134, 136]. Periods of aridity may either have led to further fragmentation due to basin geomorphology [111] or facilitated admixture as the lake levels dropped, and the available area to cichlids dwindled [60, 168]. Populations and species diverged during these repeated climatic cycles at both the regional [23] and within-lake scales [112]. Thus, climatically driven cycles of desiccation and inundation may have acted as species pumps within East African cichlids [98].

The diversification of East African cichlids also informs on the “cradle” versus “museum” dichotomy in biogeographic theory. In attempting to explain higher diversity found at lower latitudes, Stebbins [178] suggested that tropical regions may act as either “cradles,” areas with high rates of diversification, or “museums,” areas supporting diversity with low extinction rates. Given the extraordinary diversification of East African cichlids, the East African Great Lakes are clearly cradles of diversity [58]. However, the great depths of Lakes Tanganyika, Malawi, and Kivu allowed for the persistence of cichlid lineages through prolong periods of aridity [25, 28, 75, 100, 105, 136, 175]. In this way, East African lakes also conform to the “museum” hypothesis. As with a growing list of tropical species [179, 180], East African cichlids split the false dichotomy of “cradle” versus “museum” because their habitats act as both cradles and museums.

## 6. Conclusions

Fundamental to the extraordinary diversification of East African cichlids is the geologically, climatically, and ecologically dynamic environment in which they arose. Beginning at least 10–12 Ma, the western East African rift opened and created a lake basin in place of a swampy, meandering tributary to the Congo River. Seeded by Congolese cichlids, proto-Lake Tanganyika expanded and its cichlids diversified. Several of these diversifying lineages reinvaded the surrounding rivers and one lineage, the haplochromines, migrated south, perhaps via Lake Rukwa, to Lake Malawi, and north, possibly via Lake Kivu, to Lake Victoria. In each of these Great Lakes, the haplochromine cichlids formed remarkably large species flocks in an exceedingly short length of time. The evolutionary histories of the East African Great Lake cichlids were further influenced by fluctuating climatic conditions. During episodes of aridification in East Africa, the lakes were reduced in size and occasionally fully desiccated. The reduction of lake levels reshaped the lake habitats, dividing once connected populations and causing the admixture of previously isolated populations. Such processes facilitated the continued diversification of species and, at least in one instance, lead to the creation of a diverse monophyletic clade of hybrid origin. Lake Victoria most recently completely desiccated ~15 ka causing the extirpation of its endemic cichlids. As the lake infilled in the Holocene, it was then recolonized by cichlids that persisted through the arid interval in the extremely deep, but relatively small, Lake Kivu. The cichlids of Lake Kivu then went on to seed the Lake Victoria superflock, which while centered in Lake Victoria is distributed throughout the water bodies of East Africa and reaches far north into Israel via the Nile River. The cichlids of East Africa have long been recognized as an evolutionary model system in which to study phenotypic divergence and speciation. It is clear that this system also provides researchers with an exemplary system to study the impact of geologic, paleoecological, and paleoclimatic factors on the biogeography of a lineage.

## Figures and Tables

**Table 1:** Characteristics of the three great East African Lakes and their cichlid lineages.

	Lake	Lake	Lake Victoria
	Tanganyika	Malawi	
Maximum water depth (m) [54]	1470	700	79
Average water depth (m) [54]	580	264	40
Anoxic hypolimnion [54]	50–240	250	None
Surface area (km^2^) [54]	32,600	29,500	68,800
Approximate formation of lake [7, 9, 10, 55–57]	9–12 Ma	>8.6 Ma	>0.4–1.6 Ma
Approximate number of species [58]	~250	~700	~700
Number of cichlids tribes [28, 59, 60]	12–16	2	2

References in the first column refer to the table's sources.
